# Towards responsible research: examining the need for preprint policy reassessment in the era of artificial intelligence

**DOI:** 10.17179/excli2023-6324

**Published:** 2023-07-24

**Authors:** Ismail Dergaa, Karim Chamari, Jordan M. Glenn, Mohamed Ben Aissa, Noomen Guelmami, Helmi Ben Saad

**Affiliations:** 1 Primary Health Care Corporation (PHCC), Doha, Qatar; 2 Research Unit Physical Activity, Sport, and Health, UR18JS01, National Observatory of Sport, Tunis 1003, Tunisia; 3 High Institute of Sport and Physical Education, University of Sfax, Sfax, Tunisia; 4 Aspetar, Orthopedic and Sports Medicine Hospital, FIFA Medical Center of Excellence, Doha, Qatar; 5 Neurotrack Technologies, California, USA; 6 Department of Human and Social Sciences, High Institute of Sport and Physical Education of Kef, University of Jendouba, Kef, Tunisia; 7 Postgraduate School of Public Health, Department of Health Sciences (DISSAL), University of Genoa, Genoa, Italy; 8 University of Sousse, Farhat HACHED Hospital, Service of Physiology and Functional Explorations, Sousse, Tunisia; 9 University of Sousse, Farhat HACHED Hospital, Research Laboratory LR12SP09 «Heart Failure», Sousse, Tunisia; 10 University of Sousse, Faculty of Medicine of Sousse, Laboratory of Physiology, Sousse, Tunisia

## ⁯

Preprints have gained significant attraction among medical researchers as a mean of quickly sharing their preliminary and/or final findings. However, with the increased usage of preprints in scientific research, there is a growing concern about potential risks associated with this practice. Despite the advantages that preprints offer, such as facilitating rapid dissemination of research findings that contributes to promoting transparency, it is vital for the users to recognize the associated risks. Moreover, and importantly, with the emergence of advanced technologies like artificial intelligence (AI) and large language models such as Chat Generative Pre-Trained Transformer (ChatGPT) (Dergaa et al., 2023[2]), the risks associated with preprints have become even more pronounced. In this regard, this Letter to the editor aims to ***(i)*** highlight the potential risks of preprints in the growing AI era; and ***(ii)*** explore whether a complete preprint policy reassessment in academic research should be considered.

One of the primary concerns surrounding preprints is the lack of peer review process, prior to their public release. Unlike traditional journal publications, which impose rigorous peer review to manuscripts, preprints have the potential to disseminate inaccurate or incomplete information, if used incorrectly. This process could cause confusion among researchers and the general public, even resulting in serious consequences for public health. For example, during the early stages of the Coronavirus disease COVID-19 pandemic, preprints were extensively used to share research findings about the severe acute respiratory syndrome coronavirus 2 (Brierley, 2021[1]). However, many of these preprints were later found to be inaccurate, leading to widespread confusion and misinformation about the virus (Brierley, 2021[1]). Another concern is the potential for preprints to be misinterpreted or misused (Brierley, 2021[1]). Without the guidance of peer reviewers and editorial board members, information preprints may be unclearly written, leading to misunderstandings, incorrect conclusions, and potential harm. Moreover, preprints can be subject to author bias and conflicts of interest (Brierley, 2021[1]). Furthermore, researchers may be more inclined to share positive findings in preprints while suppressing negative results, leading to publication bias, which is documented to have severe implications for the scientific community and public health (Brierley, 2021[1]). While there are some potential solutions to address the risks associated with preprints, such as implementing minimal peer reviewing and establishing clear guidelines and standards, these measures are not fool proof. The original purpose of preprints was to expedite the publication process; therefore, adding peer review for preprints may not be a practical solution. Moreover, peer review is not a flawless system; time-pressure can intervene, and guidelines and standards may not always be followed consistently.

The use of AI technologies and more specifically natural language processing (NLP) models such as ChatGPT, Google Bard, Perplexity AI, has the potential to exacerbate these risks. It has been reported that ChatGPT, a powerful language model, can generate highly convincing summaries and analyses of medical research (Dergaa et al., 2023[2]). However, since ChatGPT is an AI system, it lacks the capacity for critical analysis or review, which is a crucial element in the process of scientific evaluation. Consequently, this lack of critical analysis in AI models can easily misinterpret or overstate the significance of any research, leading to misunderstandings or the dissemination of misinformation. Consequently, utilizing ChatGPT without a comprehensive understanding of the ethical considerations surrounding AI usage, particularly in the context of medical writing, poses a significant threat to academic research, particularly when sharing research as a preprint. A more substantial concern arises regarding the potential infiltration of AI-generated content into scientific papers. A study conducted in 2023 revealed that a mere 63 % of counterfeit abstracts created by ChatGPT were identified by reviewers (Else, 2023[4]). As models improve and get more robust, this reviewer identification of fakes is likely only going to get worse. Furthermore, as elucidated by Elali and Rachid (2023[3]), AI-generated research fabrication and falsification of work present significant challenges to the scientific and medical community. The ease with which authors can generate complete articles using imaginary or falsified data and publish them as preprints is a matter of concern. If we dig deeper into this scenario, imagine if ChatGPT produces a research article, posted as a preprint, proclaiming the effectiveness of a particular drug or treatment against life-threatening diseases. Such a situation could pose substantial threats to the well-being of desperate individuals, potentially leading to fatalities. This alarming prospect has the potential to undermine the integrity of scientific and medical research, akin to an uncontrolled "cancerous" growth. Similarly, a science journalist conducted a sting operation in 2013 by submitting a spurious scientific paper to open access journals, revealing that a significant number of them accepted it, even after peer review (Shaw, 2013[5]). This raised concerns about the quality control of open access journals and the potential ease of deceiving the scientific community with fraudulent papers (Shaw, 2013[5]). Although initially seen as a flaw in peer review, it is now accepted that less-skilled reviewers and advanced technology have the potential to increase the risk of fraudulent science (Shaw, 2013[5]). Hence, it is of utmost importance to enhance the quality control mechanisms in scientific publishing. Eliminating peer reviewers, who currently serve as the primary line of defense against fraudulent papers, including those generated by AI, would pose a substantial threat to the integrity of scientific research, especially within the medical domain.

Considering that ChatGPT remains an algorithm driven by machine learning, susceptible to errors and biases, it becomes imperative for readers to approach preprints with a discerning eye and engage in critical evaluation. It is advisable to seek supplementary sources of information, such as peer-reviewed publications and expert commentary, to form a well-rounded understanding of a particular topic. In the interest of prioritizing reliability and accuracy, it may be prudent for individuals to exercise caution and, whenever possible, abstain from relying solely on preprints when making decisions.

Supplementary Table 1 presents an overview of the three recommended actions aimed at mitigating the risks associated with preprints and AI technologies like ChatGPT. By implementing these actions, we can work to ensure the responsible and effective utilization of preprints and AI technologies, thereby promoting scientific progress while minimizing potential errors and risks. Furthermore, platforms that incorporate AI-generated content, should establish safeguards to guarantee the accuracy and reliability of the generated content. This may involve fact-checking procedures and human supervision to verify the information produced by the AI. It would be favorable to witness the inclusion of an "automatic generated accuracy index" alongside any AI-generated content in the future, providing readers with prior awareness of the content's reliability. Additionally, the exclusion of preprints from NLP databases like ChatGPT may reduce the generation of misleading information. Furthermore, it is crucial for researchers and journalists to receive appropriate training in the responsible use of AI technologies and to be cognizant of the associated potential risks and limitations. All stakeholders involved in the dissemination and consumption of preprints and AI-generated content bear a paramount responsibility to actively address and counteract the spread of misinformation, thus safeguarding public health.

As an alternative to preprints in academic writing, one solution is the use of a “rapid submission option” in prestigious scientific journals, but this again relies on competent and available reviewers, whose number is decreasing. This option entails editors making a preliminary decision on the manuscript within 48 hours and engaging only committed reviewers to review the manuscript within another 48 hours, for instance. However, as reviewers are not typically paid or rewarded in any way, this seems a difficult solution to implement within the current peer review framework. Therefore, compensating reviewers may serve as a motivator for them to not only accept, but complete reviews promptly. This approach offers comparable benefits to preprints and may even have some advantages. For example, published articles are likely to receive more attention and views than preprints. Additionally, the involvement of reviewers helps ensure the accuracy and transparency of the presented information. Overall, this alternative approach could be an effective means of enhancing the quality and promptness of academic writing.

Additionally, our team has proposed a reconsideration of ideas and policies, specifically suggesting a ban on citing preprint articles. To enhance efficiency and propose effective solutions, we recommend limiting access to preprint articles exclusively to journal editors and the editor-in-chief. Authors would be required to submit articles to established preprint depositories such as ChemRxiv, medRxiv, Preprints.org, Research Square, and others. From there only journal editors would have privileged access to these articles and could initiate contact with corresponding authors for potential submission. This approach is designed to achieve three primary objectives. First, it aims to expedite the publication process for authors, eliminating the delays commonly associated with the initial decision-making phase, which can range from 5 to 40 days. Second, by restricting access, we aim to reduce the occurrence of publicly facing papers containing misleading information or those generated by AI technologies like ChatGPT. Finally, this approach helps to address the shortage of papers required to complete special or regular journal issues, thereby promoting the growth and advancement of scientific journals. Ultimately, the implementation of these measures has the potential to significantly contribute to progressing the scientific field.

Considering that ***(i)*** Peer-reviewers are currently the only means of detecting AI-generated text during the publication process (with some limitations, see above), and ***(ii)*** Preprints do not undergo any type of peer review, reconsidering preprint policies is becoming urgent and mandatory to maintain the integrity of academic research. This proposal presents its challenges. Indeed, some researchers may argue that preprints are an essential tool for the rapid and open dissemination of scientific knowledge, and a complete policy reconsideration may take time and would impede innovation and progress. Nevertheless, it is essential to weigh the significant risks associated with preprints. We believe that the potential harm to public health and the scientific community far outweighs any potential benefits.

To conclude, while preprints offer many benefits, the misuse of AI technologies like ChatGPT only adds to the great risks associated with their use. Therefore, it is critical that all parties involved in medical research take a more cautious and responsible approach to the use of preprints. We suggest a complete reconsideration to the framework for publishing preprint articles. By doing so, we could ensure medical research is conducted and shared while benefiting society and minimizing the risks of “fake science” arising from the use of preprints in scientific publications.

## Declaration

### Conflict of interest

The authors declare that the research was conducted in the absence of any commercial or financial relationships that could be construed as a potential conflict of interest. 

### Authors' contributions

ID, KC and HBS: conception and design

ID, HBS, JMG and KC: analysis and interpretation of the data

ID, HBS, JMG and KC: drafting of the paper

ID, HBS, JMG, MBA, NG, and KC: revising it critically for intellectual content.

All authors gave their final approval to the version that will be published.

### Acknowledgments

Open access provided by EXCLI Journal.

## Supplementary Material

Supplementary information
