# Supplementary material for: Towards responsible research: examining the need for preprint policy reassessment in the era of artificial intelligence
Source: EXCLI J. 2023 Jul 24;22:686–9. doi: 10.17179/excli2023-6324 (PMC10471843; doi:10.17179/excli2023-6324)
Supplement: Supplementary information [file EXCLI-22-686-s-001.pdf]

EXCLI Journal 2023;22:686-689 – ISSN 1611-2156
Received: July 07, 2023, accepted: July 20, 2023, published: July 24, 2023

Supplementary information to:

Letter to the editor:

TOWARDS RESPONSIBLE RESEARCH: EXAMINING THE NEED FOR PREPRINT POLICY REASSESSMENT IN THE ERA OF ARTIFICIAL INTELLIGENCE

Ismail Dergaa[1,2,3*], Karim Chamari[4], Jordan M. Glenn[5], Mohamed Ben Aissa[6], Noomen Guelmami[7], Helmi Ben Saad[8,9,10]

1 Primary Health Care Corporation (PHCC), Doha, Qatar
2 Research Unit Physical Activity, Sport, and Health, UR18JS01, National Observatory of Sport, Tunis 1003, Tunisia
3 High Institute of Sport and Physical Education, University of Sfax, Sfax, Tunisia
4 Aspetar, Orthopedic and Sports Medicine Hospital, FIFA Medical Center of Excellence, Doha, Qatar
5 Neurotrack Technologies, California, USA
6 Department of Human and Social Sciences, High Institute of Sport and Physical Education of Kef, University of Jendouba, Kef, Tunisia
7 Postgraduate School of Public Health, Department of Health Sciences (DISSAL), University of Genoa, Genoa, Italy
8 University of Sousse, Farhat HACHED Hospital, Service of Physiology and Functional Explorations, Sousse, Tunisia
9 University of Sousse, Farhat HACHED Hospital, Research Laboratory LR12SP09 «Heart Failure», Sousse, Tunisia
10 University of Sousse, Faculty of Medicine of Sousse, Laboratory of Physiology, Sousse, Tunisia

* **Corresponding author:** Dr. Ismail Dergaa (Ph.D./ M.Sc.), Primary Health Care Corporation (PHCC), Doha, P.O. Box 26555, Qatar. E-mail: Phd.dergaa@gmail.com; idergaa@phcc.gov.qa

https://dx.doi.org/10.17179/excli2023-6324

This is an Open Access article distributed under the terms of the Creative Commons Attribution License (http://creativecommons.org/licenses/by/4.0/).

EXCLI Journal 2023;22:686-689 – ISSN 1611-2156
Received: July 07, 2023, accepted: July 20, 2023, published: July 24, 2023

Supplementary Table 1: Three actions needed to mitigate the risks associated with preprints and artificial intelligence technologies

N°	What is the action?	How and/or why?
**1.**	Researchers must:<br>• Prioritize the accuracy and reliability of their work<br>• Remain transparent about any limitations or uncertainties in their findings	This can be accomplished through:<br>• Rigorous testing and validation<br>• Clear communication of methods and data
**2.**	Peer reviewers and editors should:<br>• Exercise greater vigilance in identifying preprints in the references section of submitted manuscripts<br>• Request that authors remove preprints from references when they are identified	This is because preprints:<br>• Are not peer-reviewed<br>• May contain unverified or unreliable information<br>• The presence of preprints in a reference section could lead to incorrect or misleading conclusions
**3.**	Readers must:<br>• Approach preprints with caution<br>• Seek additional sources of information to verify the findings before making decisions	This can involve:<br>• Consulting other experts in the field<br>• Reviewing the author's reputation and track record<br>• Examining any potential biases or limitations of the research
